# Identifying Promiscuous Compounds with Activity against Different Target Classes

**DOI:** 10.3390/molecules24224185

**Published:** 2019-11-18

**Authors:** Christian Feldmann, Filip Miljković, Dimitar Yonchev, Jürgen Bajorath

**Affiliations:** Department of Life Science Informatics, B-IT, LIMES Program Unit Chemical Biology and Medicinal Chemistry, Rheinische Friedrich-Wilhelms-Universität, Endenicher Allee 19c, D-53115 Bonn, Germany; cfeldmann@bit.uni-bonn.de (C.F.); miljkovi@bit.uni-bonn.de (F.M.); yonchev@bit.uni-bonn.de (D.Y.)

**Keywords:** bioactive compounds, multitarget activity, promiscuity, polypharmacology, target classes, biological screening data, computational analysis, multiclass ligands, X-ray structures

## Abstract

Compounds with multitarget activity are of high interest for polypharmacological drug discovery. Such promiscuous compounds might be active against closely related target proteins from the same family or against distantly related or unrelated targets. Compounds with activity against distinct targets are not only of interest for polypharmacology but also to better understand how small molecules might form specific interactions in different binding site environments. We have aimed to identify compounds with activity against drug targets from different classes. To these ends, a systematic analysis of public biological screening data was carried out. Care was taken to exclude compounds from further consideration that were prone to experimental artifacts and false positive activity readouts. Extensively assayed compounds were identified and found to contain molecules that were consistently inactive in all assays, active against a single target, or promiscuous. The latter included more than 1000 compounds that were active against 10 or more targets from different classes. These multiclass ligands were further analyzed and exemplary compounds were found in X-ray structures of complexes with distinct targets. Our collection of multiclass ligands should be of interest for pharmaceutical applications and further exploration of binding characteristics at the molecular level. Therefore, these highly promiscuous compounds are made publicly available.

## 1. Introduction

Over the past decade, pharmaceutically relevant compounds with multitarget activity have been experiencing increasing attention. This has resulted from mounting evidence that the efficacy of drugs often depends on engagement of multiple targets in vivo, which is referred to as polypharmacology [1,2,3,4,5]. In this context, multitarget activity is also known as promiscuity [6]. In addition to exploring polypharmacological effects, rationalizing multitarget activity is of high interest from a basic scientific perspective. For example, one would like to better understand which molecular features might distinguish compounds with target selectivity or multitarget activity from each other, to what extent different small molecules exhibit multitarget activity, and how compounds form well-defined interactions in different binding sites. Currently, the molecular basis of multitarget activity is not well understood, although insights from X-ray structures of complexes involving promiscuous compounds are beginning to emerge [7].

The exploration of multitarget activity is complicated by the fact that apparent compound promiscuity may often be due to undesired experimental artifacts. In particular, ‘false positive’ promiscuity may result from a tendency of compounds to aggregate or react under assay conditions or engage in other non-specific interactions with different targets [8,9,10]. Therefore, in the study of multitarget activity, care must be taken to distinguish ‘true’ promiscuity from assay interference effects and other potential artifacts. This also affects computational analysis where data confidence criteria must be carefully considered and potentially liable compounds be identified [11,12]. However, the emerging ‘big data’ era in medicinal chemistry is already yielding unprecedentedly large amounts of compounds and activity data, which form a sound basis for careful promiscuity assessment, even if significant numbers of potentially questionable compounds and activity data are disregarded [12].

Three years ago, we analyzed publicly available screening data [13], identified extensively assayed compounds, and determined their activity profiles [14]. Structural analogs with large differences in their target annotations, termed promiscuity cliffs (PCs), were frequently observed. Given the large assay frequency of and assay overlap between analogs forming PCs, the presence of structural features promoting multitarget activity was confirmed. On the basis of targets from primary assays, 2070 PCs were formed involving 2158 compounds, while a corresponding analysis for targets from confirmatory assays yielded 282 PCs formed by 318 compounds [14].

In this study, we have attempted to go a step further and systematically search for highly promiscuous compounds that interact with distantly related or unrelated targets from different classes having distinct functions. Such compounds, termed herein ‘multiclass ligands’, are of particular interest both from a mechanistic promiscuity and polypharmacology perspective. This is the case because they are capable of productively interacting with different binding sites and, in addition, have the potential to interfere with distinct biological functions and pathways in vivo. In the following, our systematic analysis is reported and the results are presented.

## 2. Results and Discussion

From publicly available screening assays, extensively tested compounds were collected that qualified for rigorous promiscuity analysis. Of 341,694 compounds tested against 100 or more human targets, 125,600 were removed to avoid potential chemical liabilities. The filters applied in this step were carefully chosen to identify chemical patterns which are thought to yield assay artifacts and false positive activity assignments. The remaining 216,094 compounds were tested against 779 human targets forming ~48 million unique compound–target pairs with high-confidence activity assignments. The detailed selection criteria and corresponding assay and compound statistics are provided in Materials and Methods.

### 2.1. Qualifying Compounds and Promiscuity Degrees

For each of the 216,094 compounds, its promiscuity degree (PD) was determined as the number of different targets it was active against. The results are summarized in Table 1.

The 216,094 qualifying extensively tested compounds included nearly 130,000 (~60%) that were consistently inactive in all assays and more than 46,000 compounds that were active against a single target. A subset of 40,845 compounds (~19%) was found to be promiscuous and included 1067 compounds that were active against 10 or more targets, hence representing the most promiscuous compounds that were detected.

### 2.2. Target Distribution and Classification

**Figure 1:**
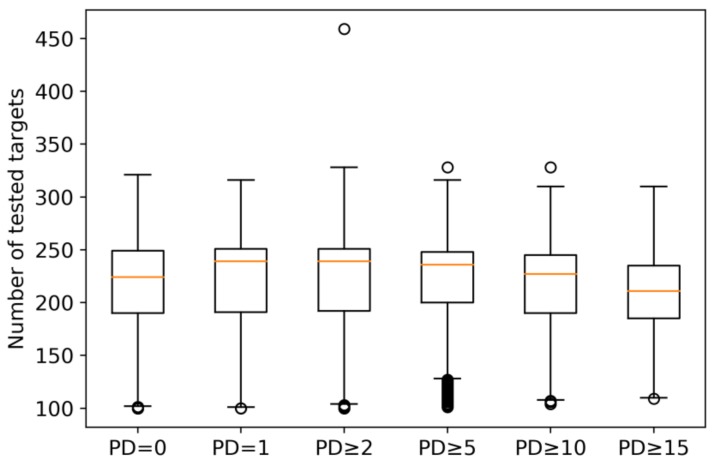
Boxplots report the distribution of assayed targets for compounds with different promiscuity degrees. PD = 0 and PD = 1 denote compounds that were consistently inactive in all assays and active against a single target, respectively. Boxplots show the smallest value (bottom line), first quartile (lower boundary of the box), median value (red line), third quartile (upper boundary of the box), largest value (top line), and outliers (points below the smallest or above the largest value).

Figure 1 reports the target distribution of compounds with increasing PD values and reveals that inactive compounds, compounds with single target activity, and increasingly promiscuous compounds were tested against overall similar numbers of targets, with median values of 211 (PD ≥ 15) to 239 (PD ≥ 2) targets per compound. Thus, most compounds were actually tested against more than 200 targets and there was no bias in target numbers for increasing PD values that might contribute to differences in observed promiscuity.

All tested proteins were assigned to major classes of drug targets, as reported in Table 2. To ensure that distinct classes of targets were considered, protein families with enzymatic functions were combined into a large class of enzymes (481 members) and G protein-coupled receptors (74) were differentiated from other receptors (13). In addition, ion channels (15) and transporters (seven proteins) were combined into two smaller classes. Minor target classes with only few members were combined into a single class (‘Others‘). Furthermore, there were a total of 109 proteins that could not be assigned to a given class (‘Unclassified‘).

Enzymes were by far the most frequent target class (~62%) in our data set derived from biological screens. For comparison, ChEMBL (version 25) [15], the major repository of compounds and activity data from medicinal chemistry, currently reports 5074 human target proteins including 1957 as enzymes (~39%). Thus, enzymes are also prevalent among targets for which compound activity data are published.

### 2.3. Promiscuous Compounds and Their Targets

**Figure 2:**
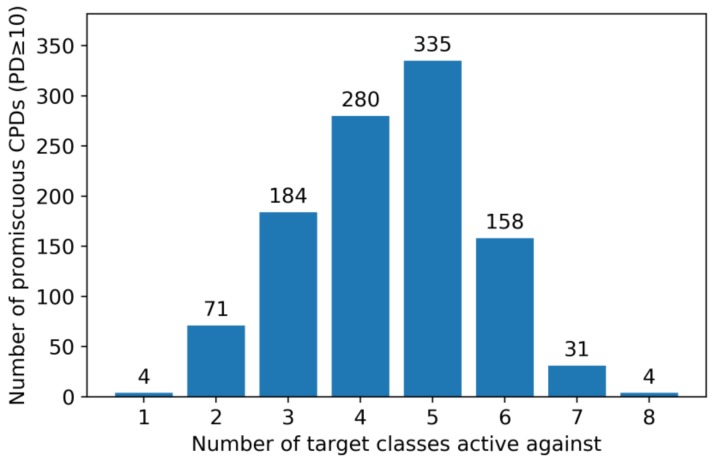
The histogram shows the distribution of highly promiscuous compounds (PD ≥ 10) over increasing numbers of target classes. Only four highly promiscuous compounds were active against 10 or more targets from a single class and four others against targets from all eight classes.

Targets of all 40,845 promiscuous compounds were classified according to Table 2. We found that 8011 promiscuous compounds (~20%) were exclusively active against multiple targets belonging to the same class, although all of them were tested against proteins from at least seven different classes. By contrast, the remaining compounds were active against targets from different classes. In particular, 1063 of 1067 highly promiscuous compounds (PD ≥ 10) displayed multiclass activity. Their target class distribution is reported in Figure 2. Most of the highly promiscuous compounds (~90%) were active against targets from three to six classes. For example, 280 compounds were active against targets from four different classes and 335 against targets from five classes. Highly promiscuous compounds with multiclass activity were focal points of our subsequent analysis and termed ‘multiclass ligands’.

### 2.4. Multiclass Ligands

**Figure 3:**
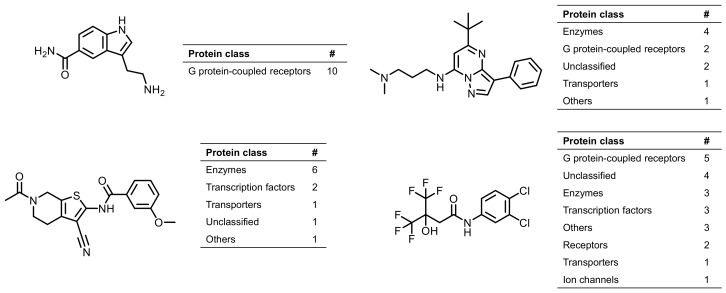
Shown are examples of multitarget ligands. For each compound, the number of targets from different classes is reported. The first ligand is exclusively active against proteins of the G protein-coupled receptor class (single class ligand), whereas the other ligands exhibited activities against targets from different classes (multiclass ligands).

The 1063 multiclass ligands we identified were promiscuous at the highest level of confidence in the context of our compound structure and activity data analysis. Moreover, these ligands best represented the paradigm of promiscuity because they were active against targets from different classes having distinct binding sites and biological functions. Furthermore, only 23 compounds did not show activity against enzymes, although all of them were tested against at least 86 different enzyme targets. Figure 3 shows exemplary multitarget ligands with activity against more than 10 targets.

### 2.5. Structure–Promiscuity Relationships

**Figure 4:**
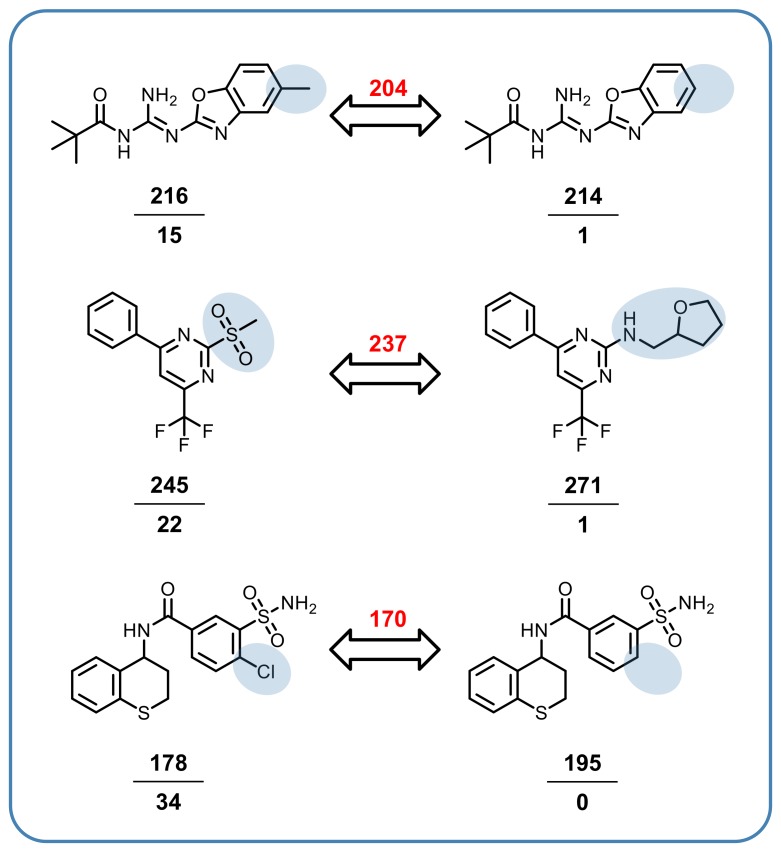
Three exemplary promiscuity cliffs (PCs) formed by multiclass ligands (left) and non-promiscuous or consistently inactive screening compounds (right) are shown. Structural modifications are highlighted. Below each compound, the number of targets it was tested (top) and active against (bottom) is given. Red numbers above arrows report shared tested targets. For example, the PC at the bottom of the figure consisted of a multiclass ligand that was tested against 178 targets and active against 34 of them and a compound that was consistently inactive against all 195 targets it was tested against. These two compounds, which were only distinguished by a chlorine substitution, were tested against 170 shared targets.

Characteristics of multiclass ligands and structure–promiscuity relationships were further explored using computational data structures designed for compound promiscuity analysis. First, a systematic search for promiscuity cliffs (PCs) involving multiclass ligands was carried out. PCs are formed by highly promiscuous compounds and weakly or non-promiscuous structural analogs. Hence, PCs reveal small structural modifications that are associated with large changes in promiscuity. In this analysis, PCs were formed between qualifying multiclass ligands (PD ≥ 10) and their single target (PD = 1) or inactive (PD = 0) analogs. Multiclass compounds that formed at least 10 PC relationships were defined as promiscuity hubs (PHs), consistent with earlier classifications. A total of 3553 PCs involving multiclass ligands were detected. Representative examples are shown in Figure 4. Analogs forming each PC were tested against large numbers of targets, ranging from 178 to 271 and in each case, the majority of targets overlapped, ranging from 170 to 245. Furthermore, the non-promiscuous or consistently inactive cliff partner was tested against a comparable or larger number of targets than its highly promiscuous analog. Thus, taken together, large numbers of assayed targets and shared targets also assigned a high level of confidence to the formation of these PCs. Here, the addition of a chlorine atom or methyl group to a non-promiscuous or inactive compound was associated with a strong increase in multitarget activity.

**Figure 5:**
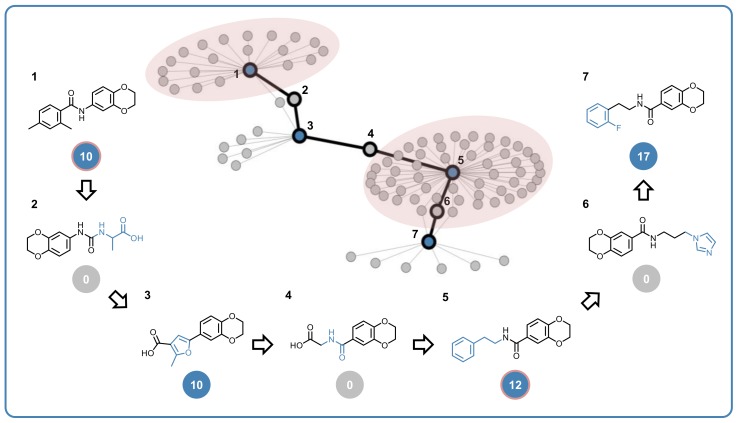
Shown is a section of the PC network formed by multiclass ligands and non-promiscuous or consistently inactive compounds. Nodes represent compounds and edges pairwise PC relationships. Blue nodes are multiclass ligands. A PC pathway formed by a sequence of alternating highly promiscuous and consistently inactive compounds is traced using a black line and the structures of pathway compounds (**1**–**7**) are displayed. Structural modifications distinguishing pairs of pathway compounds are colored blue. Below each compound, its PD value is given in a color-coded circle. The pathway contains two PHs (1 and 5) whose compound neighborhoods are highlighted.

Second, multiclass ligands were further analyzed in PC networks where nodes represent compounds and edges pairwise PC relationships. A section of the PC network with multiclass ligands is displayed in Figure 5. In the PC network, many PC pathways (PCPs) could be traced that consisted of sequences of alternating multiclass ligands and non-promiscuous analogs. Figure 5 shows a representative PCP consisting of four multiclass ligands and three consistently inactive structural analogs, highlighting chemical modifications along the path. The PCP also contained two PHs, representing densely connected multiclass ligand nodes forming a large number of PCs with non-promiscuous analogs outside the path. Accordingly, PHs and the PCs they form often suggest target hypotheses for structural analogs.

Taken together, the findings illustrated in Figure 4; Figure 5 indicated that the set of multiclass ligands we identified was rich in associated structure–promiscuity relationship information, providing many opportunities for follow-up investigations.

### 2.6. Hydrophobicity

**Figure 6:**
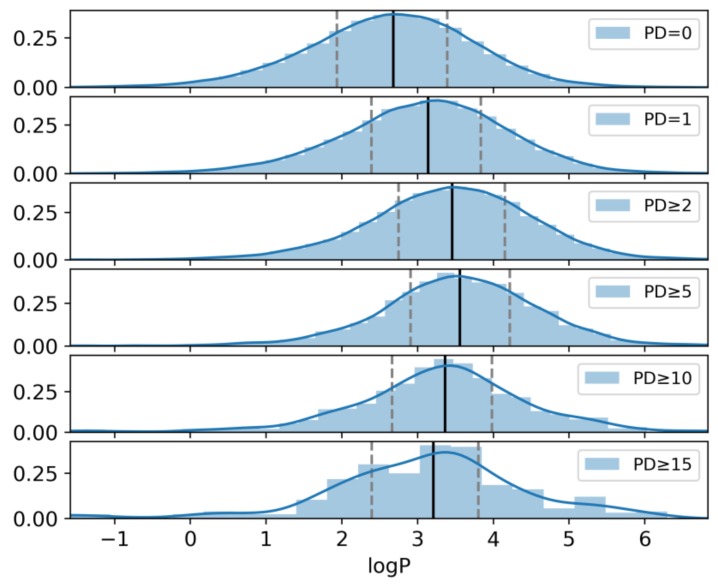
Shown is the kernel density estimation of logP values for different subsets of compounds with increasing promiscuity. In addition, for each subset, a histogram of the value distribution is shown (light blue) and the median logP value (black vertical line) and the first and the third quartile (gray vertical dashed lines) are indicated.

We have also investigated whether increasingly promiscuous compounds might be increasingly hydrophobic and significantly more hydrophobic than non-promiscuous compounds, as one might assume. Therefore, logP values were calculated for consistently inactive, non-promiscuous, and increasingly promiscuous compounds. The results are presented in Figure 6, which shows that non-promiscuous compounds and those with increasing promiscuity overall had comparable hydrophobicity, yielding similar median logP values. Hence, on the basis of logP calculations, there was no detectable trend that increasing promiscuity would correlate with hydrophobicity of compounds.

### 2.7. X-Ray Structures with Multiclass Ligands

**Figure 7:**
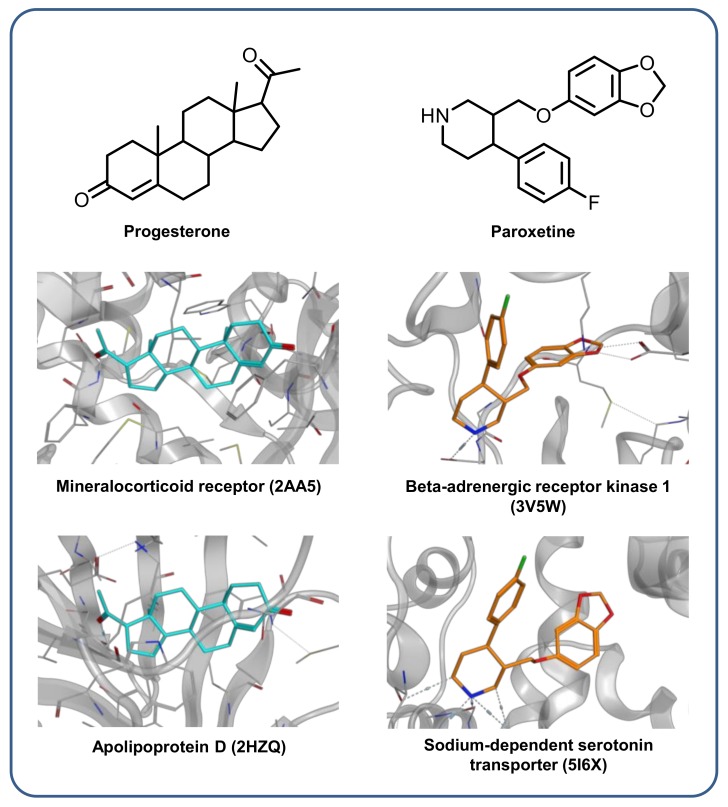
Exemplary X-ray structures of complexes of multiclass ligands with unrelated proteins. On the left, progesterone is bound to the mineralocorticoid receptor (PDB ID: 2AA5) and to apolipoprotein D (PDB ID: 2HZQ). On the right, paroxetine forms complexes with beta-adrenergic receptor kinase 1 (PDB ID: 3V5W) and the sodium-dependent serotonin transporter (PDB ID: 5I6X).

We also searched publicly available X-ray structures for complexes involving our multiclass ligands. Such structures provide direct evidence for and insights into multitarget engagement. For 34 multiclass ligands, complex X-ray structures were identified and in six cases, multiple structures with distinct target proteins were available. Two examples are shown in Figure 7 including progesterone and paroxetine, a serotonin reuptake inhibitor and marketed antidepressant. Paroxetine’s primary pharmaceutical target is the sodium-dependent serotonin transporter but it also formed a crystallographic complex with an unrelated receptor kinase. In both structures, a similar compound binding mode was observed. In addition, the progesterone example shows that multiclass ligands are not required to have any unusual synthetic structures. As a physiological steroid hormone receptor ligand with its rigid steroid framework, progesterone also formed a complex with apolipoprotein D, providing an example for a natural multiclass ligand with molecular features contrasting others such as the ligands shown in Figure 4. Hence, much remains to be learned about structure–promiscuity relationships for which multiclass ligands are thought to provide attractive starting points.

## 3. Materials and Methods

### 3.1. Biological Screening Data

Compound activity data from biological screens were extracted from PubChem BioAssay (accessed June 2017) [13]. A total of 7854 assays were obtained by applying the ‘chemical screen’ and ‘no hold’ filters to all assay sources excluding ChEMBL [15]. Assays with missing compound–target interaction data, inconsistent or unavailable gene identifiers (GIs), or multiple GIs per assay (for non-panel screens) were discarded. Applying these criteria yielded a set of 4092 single-target assays and 211 panel assays (i.e., compounds tested against multiple targets).

### 3.2. Compound Selection and Activity Assignment

For promiscuity analysis, only unambiguous qualitative compound assay results for human targets reported as ‘active’ or ‘inactive’ were considered. Potency measurements were not taken into consideration because they were generally assay-dependent (i.e., IC_50_ values) and only available for a small fraction of assays (fewer than 10%). Thus, no potency-based threshold was applicable for activity assignment on the basis of the assay data. GI numbers of assay targets were mapped to UniProt identifiers (UniProt IDs) [16]. If a single GI corresponded to multiple UniProt IDs, only a single UniProt ID with a ‘reviewed’ status was retained. Standardized compound structures were retrieved using the OpenEye OEChem toolkit [17]. In the next step, only compounds tested against at least 100 distinct human targets were selected. Applying these criteria, a total of 341,771 extensively assayed compounds were obtained. Furthermore, if multiple assays with the same target were available for a compound they were required to consistently yield the same type of interaction (active or inactive). Otherwise, the interaction was discarded. This left 341,694 compounds tested against 846 different proteins that formed ~75 million unique compound–target pairings (active or inactive). For each compound, its promiscuity degree value (PD) was calculated as the number of targets it was active against.

### 3.3. Eliminating Compounds with Potential Chemical Liabilities

To avoid consideration of compounds with potential false positive activities (assay artifacts), known pan-assay interference compounds (PAINS) [18] were detected using three public computational filters available in ChEMBL [15], RDKit [19], and ZINC [20]. Although it is not certain that designated PAINS cause false positive assay signals [21,22], they were removed since promiscuity analysis is particularly vulnerable to false positive activity assignments [12]. In addition, compounds likely to aggregate under assay conditions were eliminated using the Aggregator Advisor [8]. Furthermore, a set of 275 ‘Eli Lilly Medicinal Chemistry Rules’ [23] was applied. These empirical rules were formulated and collected over time to identify compounds that are problematic from a medicinal chemistry perspective and prone to activity artifacts. They can be divided into 17 different categories. A prominent category of rules targets potential acylating agents. Others were formulated to identify, for example, undesirable aldehydes, chelating agents, or other compounds with assay interference potential [23]. Some of the Eli Lilly rules overlap with the PAINS classification.

Computational screening for compounds with potential liabilities reduced the number of extensively assayed candidate compounds from 341,694 to 216,094. These compounds were tested against 779 targets and formed a total of ~48 million unique compound–target pairings. Prior to filtering, the compound set included 5021 highly promiscuous (PD ≥ 10) compounds. Of these, 3175 passed all three PAINS and the Aggregator Advisor filters. Applying the Eli Lilly Medicinal Chemistry Rules further reduced the number of qualifying highly promiscuous compounds to 1067 (multitarget ligands). In the context of our analysis, these compounds were promiscuous at the highest level of confidence. As a pay-off for achieving high-confidence assignments of multitarget ligands, likely false negatives were tolerated.

For non-promiscuous and promiscuous compounds, logP values based on atomic contributions [24] were calculated as a measure of hydrohobicity using RDKit [19].

### 3.4. Target Classes

Proteins were assigned to eight target classes on the basis of the ChEMBL classification scheme and keyword searching for UniProt protein and protein family names. These classes included six major categories of drug targets (Table 2). In addition, very small classes were combined to ‘Others’ and proteins that could not be assigned to drug target classes to ‘Unclassified’. In total, 1063 highly promiscuous compounds (PD ≥ 10) were defined as multiclass ligands, requiring activity against proteins from at least two target classes. The choice of the PD ≥ 10 threshold was motivated by earlier promiscuity analyses where compounds at this promiscuity level typically represented confined subsets of most promiscuous molecules [12,14].

### 3.5. Promiscuity Cliffs

Promiscuity cliffs (PCs) [25] formed by multiclass compounds and close structural analogs from biological screens were identified by systematically searching for matched molecular pairs (MMPs) with transformation size restrictions [26]. An MMP is defined as a pair of compounds that only differ by a chemical modification (transformation) at a single site [27,28]. To identify PCs, MMPs were surveyed for pairs of structural analogs with large differences between their PD values. MMPs formed by multiclass compounds (PD ≥ 10) and non-promiscuous (PD = 1) or inactive (PD = 0) analogues were defined as PCs. These selection criteria ensured that PCs studied herein were exclusively formed by highly promiscuous multiclass ligands (as discussed above) and non-promiscuous or inactive analogs. A PC network was generated in which nodes represented compounds and edges pairwise PC relationships. From PC clusters, i.e., disjoint segments of the network, exemplary PC pathways (PCPs) [29] were extracted and promiscuity hubs (PHs) [29] were identified. Following the network terminology, PHs were defined as multiclass ligands forming a higher than average number of PC relationships within the PC network. We note that the formation of PCs also illustrates the vulnerability of promiscuity analysis to compounds with false positive activity assignments. This is the case because a single false positive promiscuous compound can give rise to the formation of many erroneous PCs with weakly or non-promiscuous structural analogues.

### 3.6. X-Ray Structures with Multiclass Ligands

For multiclass ligands, a search was carried out in the Research Collaboratory for Structural Bioinformatics Protein Data Bank (RCSB PDB) [30] for complex X-ray structures containing these compounds. If complex structures with different target proteins were identified for a multiclass ligand, it was determined whether these proteins belonged to different UniProt families [31].

## 4. Conclusions

In this work, we have systematically analyzed biological screening data to search for compounds with activity against targets from different classes. Such compounds best represent promiscuous chemical entities with potential for polypharmacology. In addition, they provide interesting test cases to evaluate and better understand how small molecules can engage in well-defined interactions with distinct targets. Screening data are generally noisy. However, their use as a source for the exploration of multitarget activities has the intrinsic advantage that test frequencies can be taken into consideration, which balances the likely influence of data sparseness on promiscuity analysis. Therefore, we have first selected screening compounds that were extensively assayed against at least 100 targets. These compounds displayed a variety of activity profiles, ranging from molecules consistently inactive in all assays and others with activity against a single target to promiscuous and highly promiscuous compounds. In this context, care must be taken to eliminate compounds from promiscuity analysis that may yield assay artifacts and false positive activity readouts, which inevitably give rise to unrealistic promiscuity estimates. Therefore, currently available empirical rules from medicinal chemistry and biological screening were systematically applied to detect such compounds and exclude them from further consideration, hence ensuring rigor of promiscuity analysis. These procedures significantly reduce the number of candidate compounds and most likely give rise to false negatives since not all compounds with potentially liable features will produce artifacts. However, it is clearly preferable to tolerate false negatives over false positives, especially if large compound data volumes are available, as has been the case here. It should be considered that any molecule with interesting multitarget activity provides a candidate for follow-up investigations such that potential artifacts should be avoided as much as possible. Given our methodological framework, many promiscuous compounds were identified, but also large numbers of compounds that were inactive or only active against one or very few targets, despite large test frequencies, hence lending credence to the source data and analysis scheme. Importantly, promiscuous compounds included a subset of more than 1000 chemical entities that were active against 10 or more targets from different classes, hence meeting the ultimate goal of our study. In light of our analysis criteria, these compounds represent multiclass ligands at a high level of confidence. For exemplary compounds, X-ray structures of complexes with unrelated targets were identified, which further supported the presence of true multitarget activities. This unprecedentedly large set of high-confidence multiclass ligands provides many opportunities to further explore molecular features and interactions that trigger compound promiscuity or select template structures for polypharmacological drug design. Therefore, we make our set of multiclass ligands freely available as an open access deposition on the Zenodo platform [32]. The tab-delimited file contains the PubChem identifier (CID) for each compound, its SMILES representation, PD value, and number of targets the compound was tested against.

## Figures and Tables

**Table 1:** Reported are the numbers of compounds with different promiscuity degrees.

PD	Number of Compounds
All	216,094
PD = 0	129,215
PD = 1	46,034
PD ≥ 2	40,845
PD ≥ 5	7592
PD ≥ 10	1067
PD ≥ 15	304

**Table 2:** Reported are different target classes and the number of proteins per class.

Target Class	Number of Proteins
Enzymes	481
G protein-coupled receptors	74
Transcription factors	60
Ion channels	15
Receptors	13
Transporters	7
Others	20
Unclassified	109
